# Enhancing Flame Retardancy and Smoke Suppression in Epoxy Resin Composites with Sulfur–Phosphorous Reactive Flame Retardant

**DOI:** 10.3390/molecules29010227

**Published:** 2023-12-31

**Authors:** Xulong Ma, Ni Kang, Yonghang Zhang, Yang Min, Jianhua Yang, Daming Ban, Wei Zhao

**Affiliations:** 1 School of Chemistry and Materials Science, Guizhou Normal University, Guiyang 550001, China; 21010080287@gznu.edu.cn (X.M.); kmyo87@126.com (J.Y.); 2 Technology and Engineering Center for Space Utilization, Chinese Academy of Sciences, Beijing 100094, China

**Keywords:** smoke suppression, phosphaphenanthrene, suppressed effect, flame retardant

## Abstract

The presence of massive amounts of toxic volatiles and smoke during combustion is a very serious problem facing epoxy resin (EP) composites. Therefore, flame retardants (FRs) can simultaneously enhance flame retardancy and reduce the release of smoke and fatal gases. Herein, a novel sulfur–phosphorous reactive flame retardant (SPMS) was synthesized for epoxy resin. The high efficiency of smoke suppression and flame retardancy of the EP/SPMS-APP hybrid was investigated using a cone calorimeter, a vertical burning test, and limited oxygen index measurements. Compared with those of pure EP, the composite with 20 wt% SPMS-APP reduced the peak heat release rate (pHRR), the peak smoke production rate (SPR), and total smoke production rate (TSR) by 82%, 94%, and 84%, respectively. The results showed a remarkable suppressed effect of alleviating the fire hazard of EP using a sulfur–phosphorus flame retardant.

## 1. Introduction

Epoxy resin has been widely used in coatings, civil engineering, construction, and other fields due to its outstanding advantages such as adhesion, good mechanical properties, and chemical stability [1,2,3]. However, high flammability and massive smoke are among the main disadvantages of epoxy resins, which severely restrict their application. Therefore, fire safety EP with low smoke production urgently needs to be developed [4,5,6].

In recent decades, halogen-containing compounds have been widely utilized to optimize the inflammability of epoxy resin, but halogen-containing compounds often release toxic hydrogen halide gases, organic halides, and dioxins during combustion. Recently, in consideration of environmental aspects, several halogen-containing flame retardants have been gradually prohibited by many countries [7,8]. 

Phosphorus-based flame retardants are alternatives to halogen compounds. Several small-molecule organic-phosphorus-containing flame retardants have been widely used for EP. However, owing to their gaseous nature, these materials exhibit high flame retardancy accompanied by decreased smoke suppression, increased smoke density, or the release of corrosive gases [9,10]. Therefore, improving flame retardancy and smoke suppression performance attracted increasing attention simultaneously. There are many smoke suppressants, such as molybdenum trioxide, zinc borate, and copper oxide [11]. However, these smoke suppressants exhibit decreased efficacy because they emit water vapor mixed with smoke when used in an EP matrix [12].

With an increase in fire retardant requirements and an increase in environmental protection awareness, the hotspots of flame retardants are trending toward being halogen-free, environmentally friendly, smoke-suppressive, and having low toxicity [13]. Phosphaphenanthrene heterocyclic compounds are characterized by noncoplanarity, interactions with intramolecular or intermolecular groups, large-volume structures, and molecular polarity [14,15]. They easily bind to specific groups and retain flame retardant properties, increasing the organic solubility of new molecules [16]. The active hydrogen of phosphaphenanthrene can react with a variety of electron-deficient groups and then act as a reactive flame retardant involved in the polymerization of embedded polymer molecules [17,18,19,20,21]. Moreover, because flame retardants have a biphenyl rigid structure, the heat resistance and mechanical properties of epoxy resin may improve. Ammonium polypophosphate (APP) can play a vital role in the condensed phase, helping the matrix to form massive char residue under fire conditions and obtain a higher LOI value. It is worth mentioning that such compounds are halogen-free flame retardants, unlike halogen-based flame retardants, which can reduce environmental pollution [22].

**Scheme 1:**
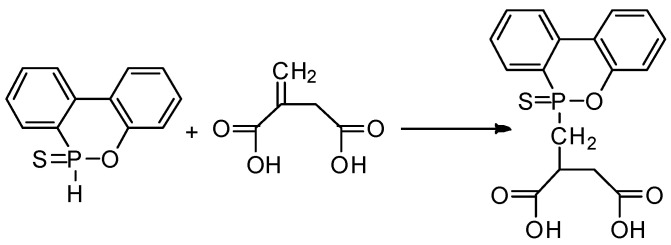
Schematic presentation of SPMS.

In this study, a phosphorous flame retardant 10-(2,5-dicarbonylpropyl) 9,10 dihydro-9-oxa-10-phosphaphenanthrene-10-sulfide (SPMS) was synthesized via a reaction between 6H-dibenzo[c,e][1,2]oxaphosphinine-6-sulfide (DOPS) and itaconic acid (ITA), as shown in Scheme 1. By incorporating SPMS-APP into EP, heat and smoke release decreased dramatically. The high efficiency of SPMS-APP in terms of smoke suppression was revealed for the first time. The smoke suppression and flame retardant mechanism of EP/SPMS-APP are discussed in detail.

## 2. Results and Discussion

### 2.1. Characterization of the SPMS

**Figure 1:**
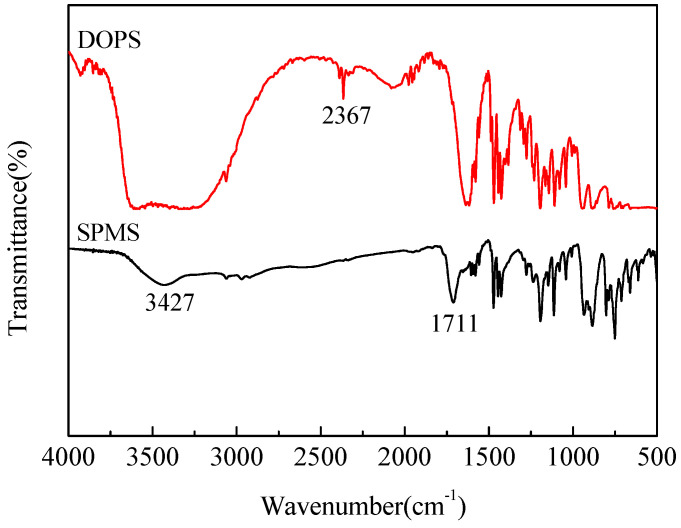
FT−IR spectra of DOPS and SPMS.

**Figure 2:**
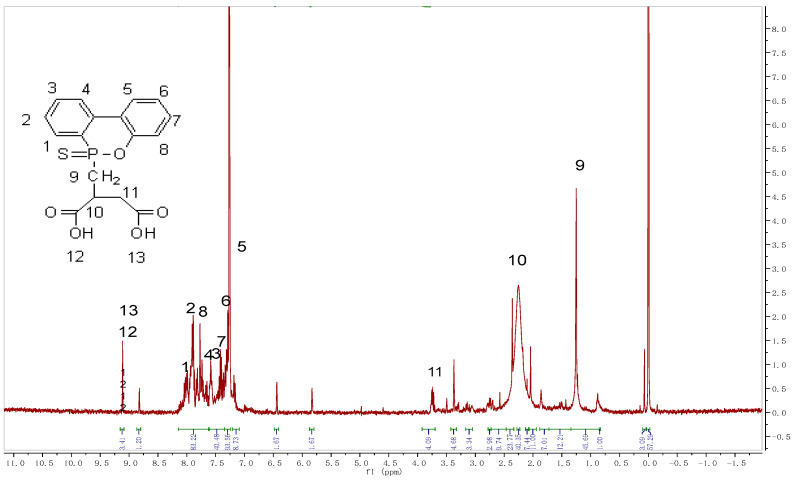
^1^H−NMR spectrum of SPMS.

The FT-IR spectra of DOPS and SPMS are shown in Figure 1. The corresponding characteristic bands were as follows: absorption at approximately 3427 cm^−1^ corresponded to --OH; -CH_2_- stretching vibration absorption appeared at approximately 2970 cm^−1^; C=O absorption at approximately 1711 cm^−1^; and absorption at approximately 1194, 1147, and 934 cm^−1^ corresponding to P-O-C(aromatic) stretching vibration. The P-C stretching vibration absorption band appeared at approximately 1472 and 1427 cm^−1^. Compared with the FT-IR spectrum of DOPS, the characteristic absorption band of the P-H bond at 2367 cm^−1^ disappeared, proving that a reaction occurred between DOPS and ITA. The ^1^H-NMR spectrum of SPMS is shown in Figure 2. ^1^H NMR (TMS, 400 MHz) δ: 9.13 (s, 2 H), 8.02 (dd, J = 14.2 Hz, J = 7.7 Hz, 1 H), 7.90 (d, J = 6.4 Hz, 1 H), 7.77 (s, 1 H), 7.74 (t, J = 7.8 Hz, 1 H), 7.59 (s, 1 H), 7.44~7.31 (m, 1 H), 7.29 (s, 1 H), 7.26 (s, 1 H), 3.74 (t, J = 7.2 Hz, 2 H), 2.25 (s, 1 H), 1.25 (s, 2 H). These results confirmed the successful synthesis of SPMS.

### 2.2. Combustion Properties of the Cured Epoxy Resins

The formulations and flame retardant properties of the EP/SPMS-APP composites are listed in Table 1.

The neat EP was highly combustible with an LOI value of 19.8% and no UL-94 grade. Moreover, the specimens were burned with flammable dripping during the test. Table 1 shows that the flame retardancy of the cured EP gradually increased with flame retardant concentration. All the LOI values of the modified EP were greater than those of the EP. Considering the same loading, EP2-5 had higher LOI values than EP-5 and EP1-5. This difference may be ascribed to the concurrent action of SPMS and APP, which acted both in the condensed and gaseous phases. Moreover, EP2-5 and EP3-10 reached UL-94 V0 ratings at 5 wt% and 10 wt% loading, respectively. Most importantly, although APP alone raised the UL-94 rating more slowly than SPMS-APP did, it helped EP achieve a higher LOI value at the same loading. Unlike EP7-20 and EP8-20, EP9-20 reached an LOI value of 34.5. 

### 2.3. Thermal Properties of Cured Epoxy Resins

**Figure 3:**
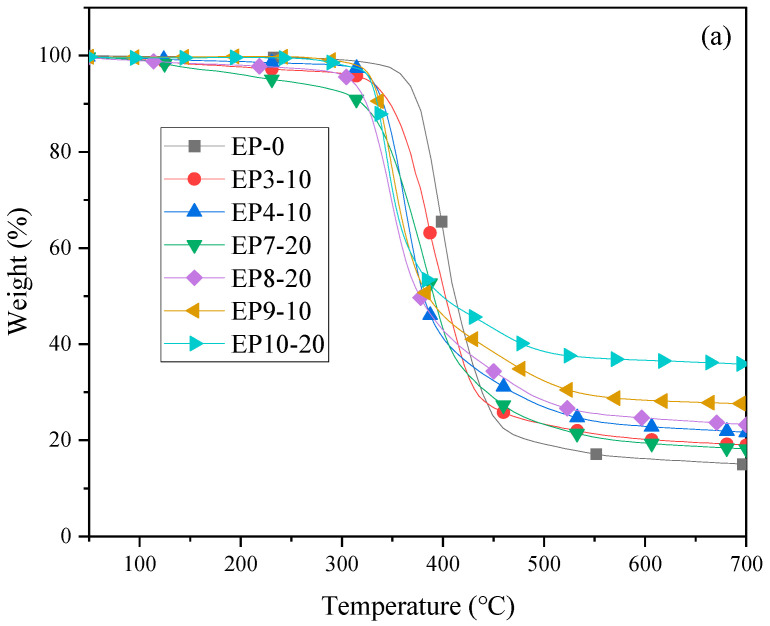

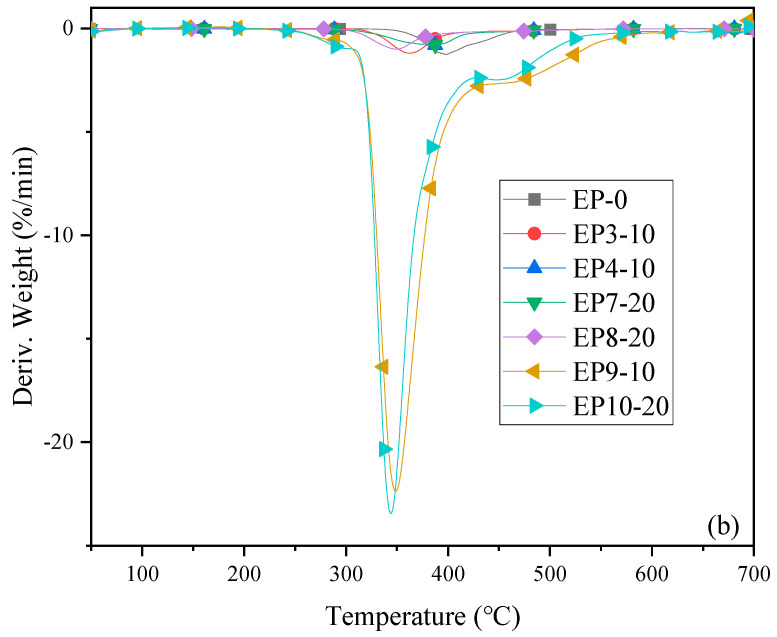
TGA (**a**) and DTG (**b**) curves of EP composites in an N_2_ atmosphere.

The thermal stability of the hybrids is shown in Figure 3. Several important parameters, including the onset degradation temperature (T_d_) of cured epoxy resins, the temperature of midpoint degradation (T_50%_), the temperature at the maximum weight loss rate (T_max_), and char residue at 700 °C, are summarized in Table 2.

The TGA curves in Figure 3 show a one-step degradation trend. With increasing FR, the Td and Tmax of EP tended to decrease under a nitrogen atmosphere, especially EP7-20, which decreased above 100 °C, which was caused by the earlier degradation of S=P-C and P-O covalent bonds. Moreover, the steric hindrance effect induced by the bulky and rigid phosphaphenanthrene group in SPMS decreased the cross-linking density of the thermoset [23]. Moreover, the flame retardant epoxy resin reached T10% and Tmax earlier than EP. Figure 3 shows that the maximum weight loss rates (R_max_) of EP0, EP3-10, EP4-10, EP7-20, EP8-20, EP9-10, and EP10-20 are 1.26, 0.97, 1.20, 0.80, 1.00, 22.37, and 23.44%/min, respectively. Considering the R_max_ values, the SPMS behaved more efficiently than the SPMS-APP, and APP alone lost weight more sharply than other samples. Therefore, the incorporation of flame retardants successfully reduced the thermal degradation rate in the low-temperature region. The epoxy resin only retained 15% of the char residues, as listed in Table 2. The char residues of EP3-10, EP4-10, EP7-20, EP8-20, EP9-10, and EP10-20 were 19, 21, 18, 23, 27 and 36%, respectively. The presence of SPMS-APP might aid in the formation of char residues, resulting in the suppression of flame spread, a reduction in flammable volatiles, and the inhibition of drip melting. APP showed high efficiency in char formation. A thick char residue layer can prevent further thermal degradation of the matrix and produce fewer inflammable volatiles.

### 2.4. Fire Hazard Analysis

**Figure 4:**
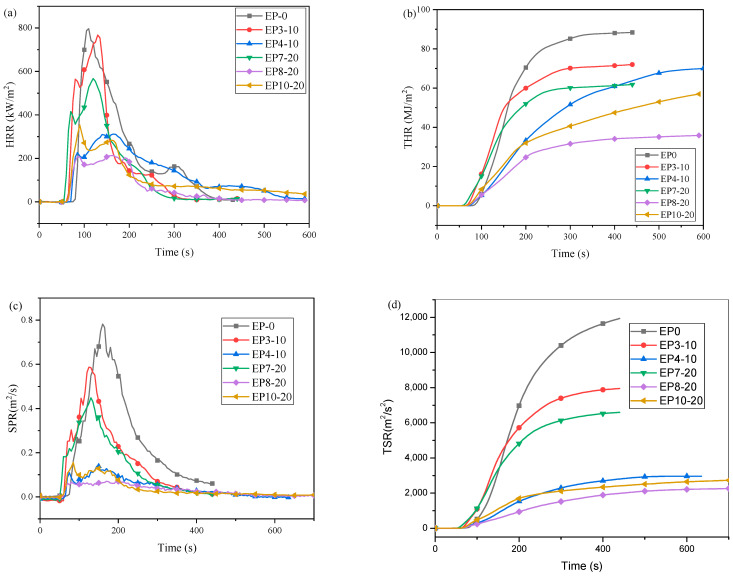
Cone calorimeter curves of the EP and SPMS/APP/EP composites: (**a**) heat release rate, (**b**) total heat release, (**c**) smoke production release, and (**d**) total smoke release.

Cone calorimeter (CC) tests were also conducted to investigate the combustion behaviors of SPMS and SPMS/APP on epoxy resin thermosets. The time to ignition (TTI), peak heat release rate (pHRR), peak smoke production rate (pSPR), total heat release (THR), total smoke rate (TSR), and fire performance index (FPI) are shown in Table 3 and Figure 4. 

The TTI was used to determine the influence of flame retardants on ignitability. As revealed in Table 3, the TTI of the pure epoxy resin was 70 s, whereas that of the flame-retarded EP composites decreased to some extent. This difference may be attributed to the early decomposition of flame retardants, which promoted degradation of the epoxy resin matrix at lower temperatures. The FPI is a parameter derived from the TTI and pHRR. The higher the values are, the better the composite behavior during fire hazard. EP8-20 had an FPI almost five times greater than that of neat EP, so it showed excellent fire performance in all tests. As shown in Figure 4a, the pHRR of the modified EP decreased over time, especially for the EP8-20 sample. 

As shown in Figure 4a,b, and Table 3, pHRR (933 kW/m^2^) and THR at 400 s (88 MJ/m^2^) were obtained for pure EP. With the addition of 20 wt% SPMS, the pHRR and THR decreased to 567 kW/m^2^ and 61 MJ/m^2^, respectively. APP alone showed in-between data between SPMS and SPMS/APP. When flame retardant SPMS was replaced by APP in sample EP8-20 at the same loading of 20 wt% SPMS/APP, the pHRR and THR decreased sharply to 164 kW/m^2^ and 34 MJ/m^2^, respectively. It can be demonstrated that SPMS/APP decomposes at low temperatures to form a protective char residue layer on the surface of the sample. These results are consistent with the results of TGA.

As shown in Figure 4c,d and Table 3, the SPR and TSR at 400 s for the EP composites decreased quickly after the incorporation of SPMS and APP. The peak SPR energy of EP7-20 was 0.44 m^2^/s, which was 43% lower than that of EP0 (0.77 m^2^/s). Moreover, the TSR at 400 s was reduced by 45%. Moreover, the samples containing both SPMS and APP had lower peak SPR values and TSR values at 400 s than those of SPMS and APP alone. The TSR at 400 s for EP4-10 was 2695 m^2^/m^2^, which was reduced by 77%. In sample EP8-20, the peak SPR and TSR at 400 s decreased dramatically, by 94% and 84%, to 0.05 m^2^/s and 1891 m^2^/m^2^, respectively. And APP alone showed a medium datum of 2341 m^2^/m^2^ between that of EP7-20 and EP8-20. The main reason may be that the suppressed effect of SPMS/APP helps greatly in promoting charring formation, and the formation of a thick carbon layer causes the number of combustible volatiles and smoke-forming materials to decrease rapidly in the gas phase during combustion. This finding implies that the application of SPMS/APP/EP composites could increase the likelihood of safe escape in cases of fire hazard due to the high FPI and reduced TSR.

### 2.5. Residual Char Analysis

**Figure 5:**
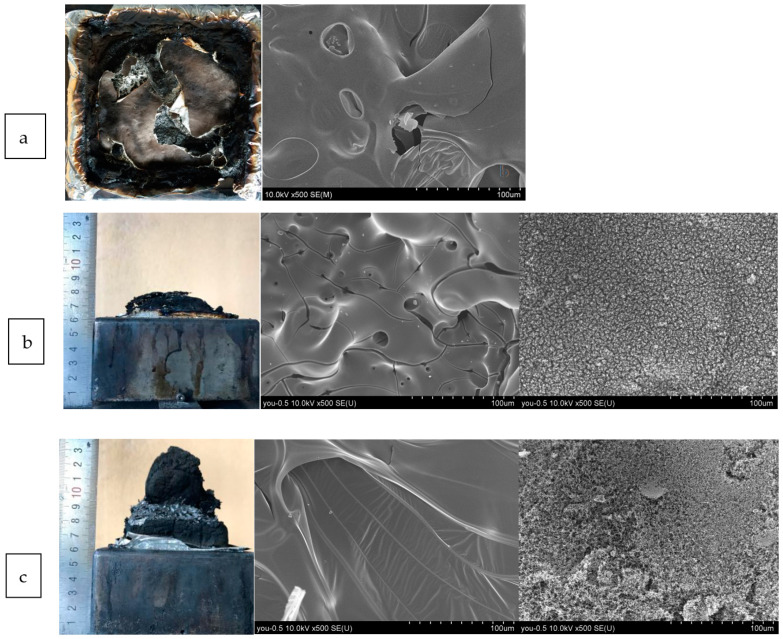

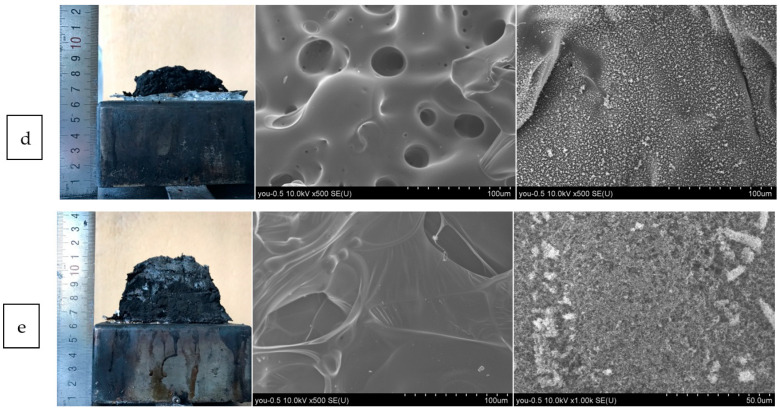
Digital, inner, and outer surface SEM images of SPMS/APP-EP after the cone test: (**a**) EP0, (**b**) EP3-10, (**c**) EP4-10, (**d**) EP7-20, and (**e**) EP8-20.

Digital photographs of char residues for the EP composites after CC are shown in Figure 5. There is a minimum quantity of char residue left behind in pure EP, which corresponds to the highest SPR, HRR, and mass loss among all the samples. Compared with EP0, EP3-10 containing only SPMS had relatively high and continuous char residue. However, there were some collapses in the char residue layer generated by the volatile compounds, and the inner surface was covered by small homogeneous bricks. As APP was incorporated into EP4-10, the amount of char residue increased significantly, and the outer surface of the char residue became increasingly compact and tough while the homogeneous bricks aggregated to form larger bricks. This revealed that APP can help char residue become more stable. It can be seen from the front view that EP4-10 and EP8-20 generated a more compact carbonaceous layer with less cracking and greater intumescence. Moreover, after combustion, the amount of residual carbon in the EP composites cured with both SPMS and APP was significantly greater than that in the EP composites cured with SPMS. The reason was that there was a suppressed effect between SPMS and APP, and the degradation of flame retardants produced a phosphorus-based acid, such as phosphoric acid, hypophosphite, and noncombustible gases, which reacted with the decomposing matrix by esterification and dehydration to promote the formation of a char layer and the noncombustible gases filled the char layer. The char layer in the molten state was expanded and foamed, and when the reaction was close to completion, the system was solidified to form a porous and foam char layer. The char layer could play a role in heat insulation and oxygen separation, so the char layer could effectively protect the polymer beneath the burn so that the amount of carbon residue was obviously increased.

### 2.6. Mechanical Properties

The influence of flame retardants on the mechanical properties of the cured EP composites was characterized via a vertical drawing test. A portion of the cured epoxy resin exhibited lower tensile strengths than did pure EP, and the tensile strength of pure EP was 39.16 MPa; however, the corresponding strength values of EP3-10 and EP4-10 decreased to 22.52 MPa and 30.94 MPa, respectively. The reason may be that EP3-10 and EP4-10 have lower phosphorous contents and rigid structures. This kind of rigid structure causes steric hindrance, and the material does not easily deform during tensile testing; moreover, a small amount of flame retardants causes stress concentration. These factors deteriorate the mechanical properties of cured EP composites.

Another part of the cured epoxy resins exhibited higher tensile strengths than pure EP. With the addition of 20 wt% SPMS/APP and SPMS to the composites, the tensile strength increased from 39.16 MPa to 49.78 MPa and 53.69 MPa, respectively. The rigid structure served as a physical cross-linking point in the cured epoxy resins, which could transfer the stress from different polymer chains to maintain the structure of the materials when a small amount of molecular chain breaks. Therefore, the tensile strength of the cured epoxy resins increased, and the flame retardants may also play a role in strengthening and toughening.

## 3. Materials and Methods

### 3.1. Materials

6H-dibenzo[c,e][1,2]oxaphosphinine-6-sulfide (DOPS) was synthesized in our laboratory [24]. Epoxy resin (DGEBA E-44), a commercial product, was purchased from Blue Star Chemical New Materials Co., Ltd. (Beijing, China). Itaconic acid (ITA) was supplied by Sinopharm Group Chemical reagents, Shanghai, China. Tetrahydrofuran (THF) and acetone were purchased from Tianjin Fuyu Fine Chemical Co., Ltd. (Tianjin, China). APP was obtained from Shifang Taifeng New Flame Retardant Co., Ltd. (Chengdu, China). M-phenylenediamine (PDA) was purchased from Tianjin Guangfu Fine Chemical Research Institute (Tianjin, China). All chemicals were used as received and without further purification.

### 3.2. Synthesis of SPMS

The schematic route of SPMS is illustrated in Scheme 1, and the details are as follows: 3.1 g of DOPS, 1.83 g of ITA, and 60 mL of THF were introduced into a four-neck 250 mL flask equipped with a nitrogen inlet and a mechanical stirrer. The resulting mixture was heated to reflux with agitation under a nitrogen gas atmosphere for 5.5 h. After cooling to room temperature, the precipitate was removed by filtration; the resulting material was subsequently dried and recrystallized. A white solid of SPMS was obtained with a yield of 75.7% and a m.p. of 178~181 °C. 

### 3.3. Preparation of the EP/SPMS-APP Hybrid

The cured EP was prepared via a thermal curing process. Briefly, the epoxy resin was heated to melt first. Subsequently, flame retardants were added separately according to the mass ratio in Table 1, and the mixture was sufficiently stirred. The mixture was subsequently placed in a preheated vacuum oven to degas at 80 °C. After that, PDA was added accurately under vigorous stirring until it was completely dissolved before being poured into the mold. The mixture was subsequently cured in an air-drying oven at 80 °C for 2 h, followed by 100 °C for 2 h, and then postcuring at 120 °C for 2 h. Thereafter, the curd EP was permitted to cool slowly to room temperature. Then, the splines and trimmed parts were removed so that they could meet the requirements of the corresponding performance test.

### 3.4. Characterization

The limiting oxygen index (LOI) was measured on a JF-5 oxygen index instrument (Jiangning Analytical Instrument Factory, Nanjing, China) according to the ASTM D-2863 testing procedure, with sample dimensions of 80 × 6.5 × 3.2 mm^3^. The vertical burning test (UL-94) was performed on a CZF-6 instrument (the same factory used for JF-5) according to the ASTM D-3801, with sample dimensions of 125 × 13 × 3.2 mm^3^. A mechanical performance test was carried out using a universal testing machine (Jinan East Special Equipment Co., Ltd. Jinan, China) according to the GB T13022-91 testing procedure, with a stretch rate of 1 mm/min. Thermal gravimetric analysis (TGA) was performed with a thermal analyzer (NETZSCH STA 409 PC/PG, Bavaria, Germany) at a heating rate of 20 °C/min from 20 °C to 700 °C under a nitrogen flow of 60 mL/min. Cone calorimeter measurements were performed according to the ISO5660-1 protocol at an incident flux of 35 kW/m^2^, with sample dimensions of 100 × 100 × 1.2 mm^3^. Scanning electron microscopy (SEM) images of the inner and outer surfaces of the char residue after CC were obtained by using a Hitachi S-4800. Char layers were sputter coated with a thin layer of gold, and SEM was performed at an accelerating voltage of 10 kV.

## 4. Conclusions

In this article, a novel phosphorous-containing flame retardant, SPMS, was successfully synthesized and characterized. The flame retardants in the epoxy resins exhibited excellent flame retardancy and intumescent effects. The flame retardancy was obviously improved, and the cured epoxy resins had higher char yields, which showed that there was a concurrent action of the SPMS and APP. The CC results showed that the pHRR, av-HRR, THR, and TSR values of the epoxy resins decreased significantly. This kind of reactive flame retardant is promising for other polymer matrices that interact with diacid functional groups or react with other functional group flame retardants.

## Figures and Tables

**Table 1:** Formulations and flame retardancy of epoxy resin thermosets.

Sample	Mass Fraction (%)			LOI (%)	UL-94
	EP + PDA	SPMS	APP		
EP0	100	0.00	0.00	19.8	N.R.
EP-5	95	0.00	5.00	21.4	N.R.
EP1-5	95	5.00	0.00	21.1	V-1
EP2-5	95	1.67	3.33	20.8	V-0
EP3-10	90	10.0	0.00	21.5	V-0
EP4-10	90	3.33	6.67	21.7	V-0
EP5-15	85	15.0	0.00	24.7	V-0
EP6-15	85	5.00	10.0	26.4	V-0
EP7-20	80	20.0	0.00	25.0	V-0
EP8-20	80	6.67	13.33	27.3	V-0
EP9-10	90	0.00	10.0	26.8	V-0
EP10-20	80	0.00	20.0	34.5	V-0

**Table 2:** Related TGA data for EP composites.

Sample	T_d_ (°C)	T_50%_ (°C)	T_max_ (°C)	R_max_ (%/min)	R_700_ (%)
EP0	357	408	398	1.26	15
EP3-10	324	401	382	0.97	19
EP4-10	329	379	363	1.20	21
EP7-20	233	389	387	0.80	18
EP8-20	306	375	348	1.00	23
EP9-10	330	385	349	22.37	27
EP10-20	328	399	344	23.44	36

**Table 3:** Cone calorimeter data for the flame-retarded EP composites.

Sample	TTI (s)	pHRR (kW·m^−2^)	pSPR (m^2^·s^−1^)	THR at 400 s (MJ·m^−2^)	TSR at 400 s (m^2^·m^−2^)	FPI (s·m^2^·kW^−1^)
EP0	70	933	0.77	88	11,640	0.075
EP3-10	59	767	0.58	71	7880	0.076
EP4-10	65	311	0.14	70	2695	0.20
EP7-20	48	567	0.44	61	6529	0.084
EP8-20	60	164	0.05	34	1891	0.36
EP9-20	59	354	0.16	47	2341	0.167

## Data Availability

Data are contained within the article.
